# Type I error rates of rare single nucleotide variants are inflated in tests of association with non–normally distributed traits using simple linear regression methods

**DOI:** 10.1186/s12919-016-0060-7

**Published:** 2016-10-18

**Authors:** Tae-Hwi Schwantes-An, Heejong Sung, Jeremy A. Sabourin, Cristina M. Justice, Alexa J. M. Sorant, Alexander F. Wilson

**Affiliations:** Genometrics Section, Computational and Statistical Genomics Branch, National Human Genome Research Institute, National Institutes of Health, Baltimore, MD 21224 USA

## Abstract

In this study, the effects of (a) the minor allele frequency of the single nucleotide variant (SNV), (b) the degree of departure from normality of the trait, and (c) the position of the SNVs on type I error rates were investigated in the Genetic Analysis Workshop (GAW) 19 whole exome sequence data. To test the distribution of the type I error rate, 5 simulated traits were considered: standard normal and gamma distributed traits; 2 transformed versions of the gamma trait (log_10_ and rank-based inverse normal transformations); and trait Q1 provided by GAW 19. Each trait was tested with 313,340 SNVs. Tests of association were performed with simple linear regression and average type I error rates were determined for minor allele frequency classes. Rare SNVs (minor allele frequency < 0.05) showed inflated type I error rates for non–normally distributed traits that increased as the minor allele frequency decreased. The inflation of average type I error rates increased as the significance threshold decreased. Normally distributed traits did not show inflated type I error rates with respect to the minor allele frequency for rare SNVs. There was no consistent effect of transformation on the uniformity of the distribution of the location of SNVs with a type I error.

## Background

Recent advances in sequencing technologies have made it more affordable to sequence whole exome data. In next-generation sequencing data, the proportion of rare variants (minor allele frequency [MAF] < 0.05) is substantially larger than the proportion of more common variants (MAF ≥ 0.05) typically used in genome-wide association studies (GWAS). However, these rare sequence variants present a challenge because there are often too few rare alleles for traditional statistical tests, making it more difficult to identify rare variants that are associated with the trait of interest. Also, the increased density of next-generation sequence variants makes it difficult for traditional methods to identify independent associations in a region of interest because of multicollinearity.

Although it is known from statistical theory that comparing error rates from non-normal distributions to normal distributions results in inflation of type I error [1, 2], the specific role of the frequency of the minor allele with respect to type I error in this situation is not clear. Tabangin et al. [3] reported that rare single-nucleotide polymorphisms (SNPs) did not show an increased type I error rate for tests of association, although they did note that there was an increase in type I error rate at a critical value of 10^−4^. In this study, we used the Genetic Analysis Workshop (GAW) 19 whole exome sequence data [4] on unrelated samples to explore the effects on the average type I error rate of the MAF of the single nucleotide variants (SNVs, defined here as variants without constraints on the MAF) for different null trait distributions and critical values.

Furthermore, Papanicolaou et al. [5] noted an increase in the type I error rate for short tandem repeat polymorphisms (STRPs) at the telomeres in linkage analysis. The distribution of the physical position of SNVs was also investigated in an attempt to confirm or refute this finding.

## Methods

### Genotype data

VCFtools [6] was used to obtain alternative allele counts (NALTT field) for each biallelic SNV from the odd-numbered chromosomes for the 1943 unrelated samples. Alternative allele counts were converted to 2-allele genotype calls. The MAF for each SNV was calculated with PLINK [7]. All monomorphic SNVs (MAF = 0) and SNVs with greater than 5 % missing were excluded, leaving 313,340 SNVs for analysis.

### Trait data

To investigate the average type I error rate, 2 quantitative traits were simulated under the null hypothesis of no genetic effect: one from a standard normal distribution (with mean 0 and variance 1) and one from a gamma distribution, using the “rgamma” function in R with shape parameter 3 and scale parameter 20. In addition, 2 transformations were performed on the gamma-distributed trait to satisfy the normality assumption in regression analysis: the log_10_ transformation and the rank-based inverse normal transformation (RIT). Trait Q1, provided by GAW 19, was also tested. A total of 200 replications for each of these 5 null traits were generated.

### Statistical analysis

**Fig. 1:**
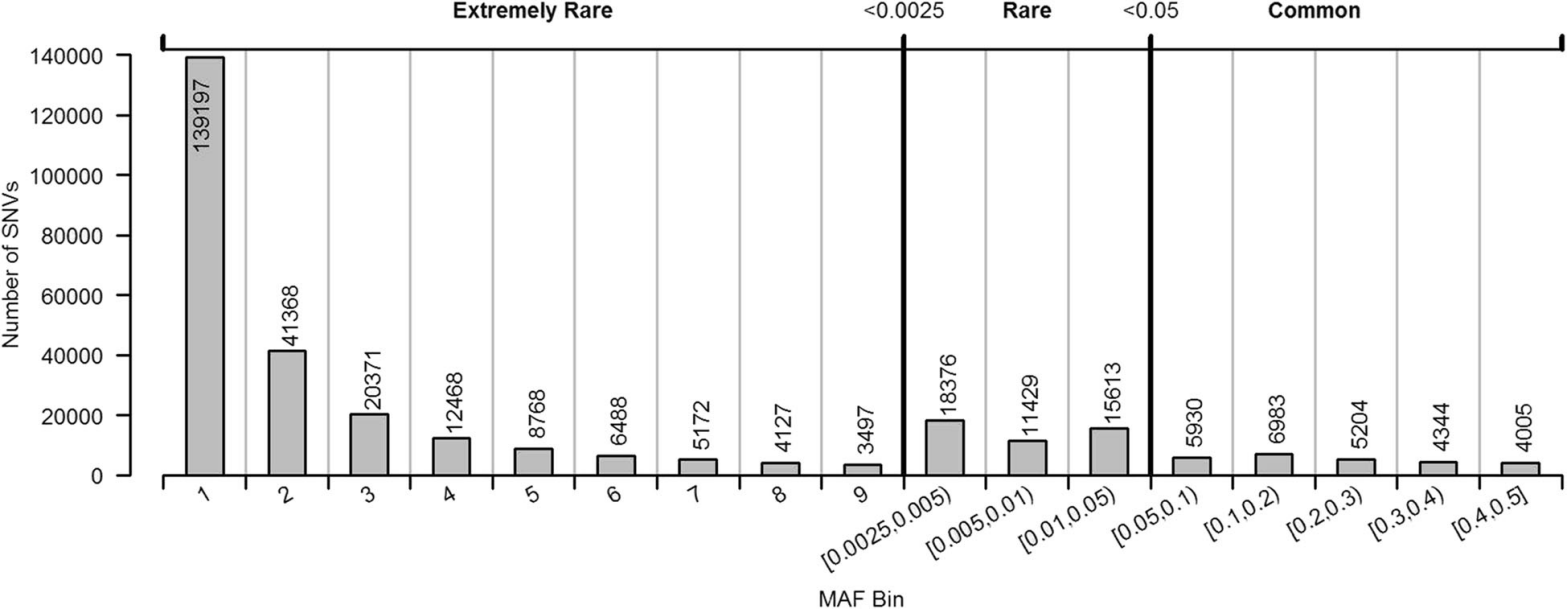
Frequency of SNVs by MAF

**Fig. 2:**
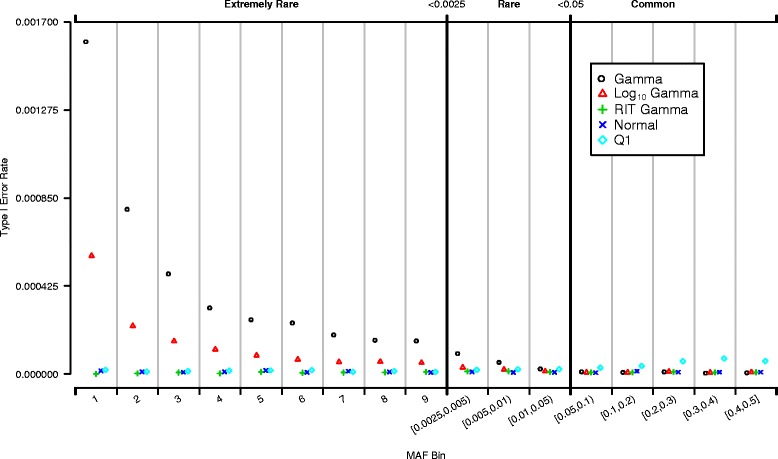
Distribution of type I error rate by MAF. Type I error rate versus MAF. Different color/symbols indicate different null traits. Each point indicates average type I error rate at the critical level of 10^-5^ of SNVs grouped by MAF; extremely rare variants (MAF < 0.0025) are classified by number of occurrences of the rare allele, while rare (0.0025 ≤ MAF < 0.05) and common (MAF ≥ 0.05) ones are classified by MAF range

Tests of association between each SNV and each null trait were determined with simple linear regression as implemented in PLINK [7]. Type I error rates were estimated with all the SNVs in a specified MAF class as shown in Figs. 1 and 2. The minimum number of observations for the determination of average type I error rate was 200 replications times the 3497 observations in the smallest class (699,400 observations). Critical values of both 0.001 and 10^-5^ were considered as thresholds for defining a type I error.

### Tests of the uniformity of the distribution of the locations of the single nucleotide variants with any type I error

Two Chi-squared goodness of fit tests were used to determine whether the SNVs with any type I errors in the 200 replicates were uniformly distributed. The uniformity of type I errors was tested among groups defined as (a) chromosomes and (b) 10 Mb intervals.

## Results

Table 1 summarizes the observed mean, variance, skewness, and kurtosis averaged over 200 replicates of each trait used in this study.

**Table 1:** Estimates^a^ of the mean, variance, skewness, and kurtosis of each simulated trait

Distribution	Mean	Variance	Skewness	Kurtosis
Normal	−0.003	1.00	−0.001	3.00
Gamma	59.97	1197.63	1.15	4.97
Log_10_ gamma	1.70	0.07	−0.62	3.77
RIT^b^gamma	0.0	1.00	0.0	2.98
Q1	41.34	113.36	−0.03	2.99

^a^These values are averages over 200 replicates for each trait

^b^Rank-based inverse normal transformation

Figure 1 summarizes the SNVs by MAF. Extremely rare SNVs were defined as any SNV with MAF less than 0.0025 and were categorized by counts of the minor allele for these classes. There were a total of 241,456 extremely rare SNVs (77 % of all SNVs considered), and more than half of those occurred only once per sample. Rare SNVs were defined as those with MAF between 0.0025 and 0.05 and common SNVs as those with MAF greater than 0.05. Rare and common SNVs were categorized by MAF range.

### Type I error rate vs. minor allele frequency of the single nucleotide variant, the degree of departure from normality of the trait, and the critical value

Figure 2 shows the type I error rates for a critical value of 10^-5^ by MAF of the SNVs for all 5 traits. There was inflation of type I error rate for a given critical level based on the MAF of the SNVs and on the degree of departure from normality of the trait. For the non–normally distributed traits (gamma and log_10_-transformed gamma), there was a substantial inflation of type I error rate for rare and extremely rare SNVs (MAF < 0.05), but not for common SNVs (MAF ≥ 0.05); the type I error rates increased as the MAF decreased. The inflation of the type I error rate was greatest for the gamma trait and somewhat smaller for the more normally distributed log_10_-transformed gamma. The type I error rates for the normal and rank-based inverse normal transformed gamma traits were not inflated and had the nominal type I error rate (10^-5^). Inflation was greatest for the gamma trait for singleton SNVs, more than 150 times the nominal level. However, the amount of inflation depended on the critical value used. For example, with the critical level of 10^-3^ the type I error rate for the gamma trait for singleton SNVs was 8 times the nominal value (results not shown). It is important to note that there was no inflation of type I error rates for normally distributed traits at any critical level, regardless of the MAF. Trait Q1 was nearly normally distributed, and it did not show inflated type I error rate for rare or extremely rare SNVs. However, type I error rates for common SNVs were higher than expected.

### Tests of uniformity of the distribution of type I errors

Table 2 displays the *p* values for both tests for each of the 5 traits with type I errors defined using a 10^-3^ threshold. Overall, the results were neither consistent nor conclusive in terms of the transformation.

**Table 2:** *P*-values for goodness of fit tests of uniformity of the distribution of SNVs with any type I error

Test among	Normal	Gamma	Log_10_ gamma	RIT gamma	Q1
Chromosomes	0.113	0.880	0.338	0.006	0.008
10 Mb intervals	0.234	0.966	0.088	0.001	0.007

## Discussion

In this study, the effects the MAF of the SNVs, the degree of departure from normality of the trait and the position of the SNVs on type I error rates were investigated on 5 simulated “null” traits, each with 200 replicates, and the genotypes from the GAW19 whole exome sequencing data in the unrelated samples.

Observed type I error rates for rare and extremely rare SNVs (MAF < 0.05) for non–normally distributed traits (gamma and log_10_-transformed gamma) increased over the nominal level with increasing departure from normality, with decreasing MAF of the SNVs and with decreasing critical level. However, observed type I error rates for normally distributed traits were close to the nominal level regardless of the MAF of the SNVs. Trait distributions with differing degrees of departure from normality made a substantial difference in the type I error rate for the test of association with simple linear regression with rare SNVs. The gamma-distributed trait showed the largest differences between observed and expected type I error rates. When the gamma trait was log_10_-transformed to be more normal, the difference became smaller. When a more extreme transformation (RIT) was used, the trait was effectively normally distributed and did not show inflated type I error. This indicates that transforming non–normally distributed traits helps to control type I error rate. No inflation of type I error rate was observed for common SNVs (MAF ≥ 0.05) for the 2 non–normally distributed traits considered (gamma and log_10_-transformed gamma).

Trait Q1 behaved similarly to the normally distributed trait in that it did not produce increased type I error rate among rare and extremely rare SNVs. Unlike the other tested traits, however, Q1 showed a slightly higher than expected type I error rate for the common variants (MAF ≥ 0.05). Trait Q1 was generated under a different null hypothesis from the other 4 traits that were simulated for this study.

The results of the tests of uniformity of the distribution of the SNVs with type I errors showed no obvious positional effect with respect to trait transformation. It is relevant to note that Papanicolaou et al. [5] reported increased type I error in the telomeres with Haseman-Elston linkage analyses using STRPs; however, the differences for association tests were minimal. The results from this study likely corroborate the association data, but the exome data had poor coverage of the telomeres, limiting what can be inferred.

## Conclusions

In summary, both rare and extremely rare SNVs produced more type I errors than the nominal rate for traits with departures from normality. This effect was ameliorated by transforming the trait to be more normal. Common variants seemed to be protected from this increase in type I error for most of the tested traits.
